# Reframing Patient and Parental Experience in Pediatric Healthcare: Medical Gaslighting as a Diagnostic Safety Vulnerability

**DOI:** 10.3390/children13080981

**Published:** 2026-07-24

**Authors:** Susmitha Nagula, Nasreen Ahmed, Kryss Shane, Anthony D. Slonim

**Affiliations:** 1 PGY 2, Department of Pediatrics, Virginia Tech-Carilion School of Medicine, Roanoke, VA 24014, USA; snagula@carilionclinic.org (S.N.); nahmed2@carilionclinic.org (N.A.); 2 National University, San Diego, CA 92123, USA; 3 Fordham University, New York, NY 10458, USA; 4 Departments of Internal Medicine and Pediatrics, Carilion Clinic, Virginia Tech-Carilion School of Medicine, Roanoke, VA 24016, USA

**Keywords:** medical gaslighting, diagnostic safety, pediatric diagnosis, diagnostic error, cognitive bias, epistemic injustice, patient safety, diagnostic reasoning

## Abstract

Medical gaslighting has emerged as a widely used term describing situations in which patients or caregivers perceive that their symptoms are minimized, dismissed, or prematurely attributed to psychological causes without adequate clinical evaluation. Although the term has gained prominence through patient narratives and public discourse, many of the underlying mechanisms correspond to well-established contributors to diagnostic error, including cognitive bias, communication failures, and diagnostic overshadowing. In pediatric care, these challenges are amplified because clinicians frequently rely upon caregiver-mediated histories while children may have a limited ability to communicate evolving symptoms. This narrative review examines medical gaslighting through the framework of diagnostic safety and explores how epistemic injustice, cognitive bias, and failures in information gathering and interpretation may contribute to delayed, missed, or incorrect diagnoses. Relevant literature was identified through a structured narrative review of publications addressing diagnostic error, cognitive bias, epistemic injustice, diagnostic safety, and pediatric communication. The literature was synthesized conceptually to develop an integrated framework linking patient experiences with established diagnostic safety models. We propose that reframing medical gaslighting as a diagnostic safety vulnerability rather than an allegation of clinician misconduct provides opportunities for measurable quality improvement through enhanced communication, structured reassessment, cognitive debiasing strategies, and integration of patient- and caregiver-reported information into diagnostic reasoning.

## 1. Introduction

Medical gaslighting describes situations where patients or caregivers perceive that their symptoms are being minimized, dismissed, or prematurely attributed to psychological causes without adequate clinical evaluation [1]. While the term has gained prominence in public discourse, it does not represent a new clinical phenomenon. Instead, it reflects long-standing diagnostic safety challenges including cognitive bias, diagnostic overshadowing, communication breakdowns, and variability in how patient and caregiver information is incorporated into clinical decision making [2,3,4].

Diagnostic error remains a major and persistent threat to patient safety worldwide [5,6,7]. These errors frequently arise under conditions of diagnostic uncertainty, where clinicians must integrate evolving clinical information while balancing competing diagnostic hypotheses. In these contexts, patient- and caregiver-reported symptoms represent essential diagnostic data. When these reports are discounted, inadequately explored or integrated, or prematurely attributed to non-organic causes, diagnostic delay or error may occur.

In pediatric medicine, these challenges may be amplified because diagnostic reasoning often depends on caregiver observations, developmental stage, and communication abilities. Symptom reporting is frequently indirect, requiring clinicians to synthesize information from both the child and caregivers while accounting for evolving disease presentations. Consequently, failures in communication or diagnostic reasoning may have disproportionate effects on diagnostic accuracy and patient safety in this vulnerable population.

Recent patient safety frameworks, including the National Academy of Medicine and the Emergency Care Research Institute (ECRI) reports, highlight failures to recognize and incorporate patient and caregiver concerns as important contributors to diagnostic error and preventable harm [5,8]. Medical gaslighting may arise from multiple human factors including cognitive biases, communication failure, and organizational pressures, but we conceptualize it as a diagnostic safety vulnerability because it represents a recurrent failure mode through which clinically relevant patient and caregiver information is discounted.

In this review, medical gaslighting is used as a descriptive heuristic rather than an attribution of malicious intent by clinicians. It reflects how patients experience failures in diagnostic reasoning rather than implying deliberate attempts to deceive or invalidate patients. Although the term medical gaslighting may be interpreted as implying intentional or malicious behavior, we use it to describe the patient or caregiver experience of having legitimate concerns repeatedly discounted or dismissed, regardless of clinician intent. This phenomenon overlaps with, but is distinct from, concepts such as diagnostic overshadowing, medical invalidation, inappropriate dismissal of concerns, and medical paternalism.

The aims of this review are to synthesize diagnostic safety literature, to clarify how medical gaslighting can be understood as a manifestation of diagnostic process failure and to identify potential system-level interventions to improve diagnostic care in pediatrics. Importantly, this work does not propose medical gaslighting as a new diagnostic construct, but as a patient-centered descriptor of existing diagnostic safety failures.

## 2. Methods

This manuscript is a narrative review and conceptual synthesis. Its objective was to synthesize current evidence relating to medical gaslighting, diagnostic safety, cognitive bias, epistemic injustice communication science, and pediatric diagnostic reasoning to develop an integrated conceptual framework.

A structured literature search was performed using PubMed, Scopus and Google Scholar. Search terms included combinations of ‘medical gaslighting’, ‘diagnostic error’, ‘diagnostic safety’, ‘cognitive bias’, ‘diagnostic overshadowing’, ‘epistemic injustice’, ‘patient safety’, ‘pediatric diagnosis’ and ‘clinical communication’. Reference lists of relevant articles and major patient safety reports were also reviewed to identify additional publications.

Included literature consisted of peer-reviewed original research, review articles, conceptual papers, and major consensus reports relevant to diagnostic reasoning and patient safety. Emphasis was placed on publications from the National Academy of Medicine, ECRI, and foundational literature addressing cognitive bias, diagnostic error, and epistemic injustice.

Because of the heterogeneous nature of the available evidence and the conceptual objective of this manuscript, findings were synthesized narratively rather than through quantitative meta-analysis. This review was intended to generate an integrated conceptual framework rather than to estimate pooled effect sizes. Accordingly, this manuscript follows accepted principles for narrative reviews and is not presented as a systematic review.

## 3. Results

### 3.1. Reframing Medical Gaslighting as a Diagnostic Safety Problem

Diagnostic error remains a persistent and measurable patient safety problem across healthcare systems [6,7]. The rapid emergence of the term *‘medical gaslighting’* reflects a perceived gap between patients’ lived experiences and traditional descriptions of diagnostic error. For many clinicians, the phrase may appear imprecise or accusatory; however, for patients and caregivers, it frequently captures the experience of not being heard during periods of diagnostic uncertainty.

Reframing these experiences within established diagnostic safety frameworks helps to bridge the conceptual gap by translating lived patient experiences into observable diagnostic processes that can be evaluated, measured, and improved.

This perspective aligns closely with the National Academy of Medicine’s diagnostic safety framework, which identifies failures in information gathering, interpretation, communication and follow-up as major contributors to diagnostic error [5]. Similarly, the 2025 ECRI report identified dismissal of patient and caregiver concerns as one of the leading patient safety threats [8]. These failures frequently occur under conditions of uncertainty, time pressure, fragmented care, and reliance on heuristic reasoning [2,3,4].

In pediatric practice, these vulnerabilities may be amplified because clinicians frequently depend upon caregiver-mediated histories while simultaneously interpreting developmental variability, evolving symptom patterns, and limited objective findings. Consequently, medical gaslighting remains relatively underexplored within pediatric diagnostic safety despite directly affecting how patient- and caregiver-reported information is incorporated into diagnostic reasoning.

In this review, medical gaslighting is conceptualized as a diagnostic safety failure mode rather than evidence of provider misconduct. The phenomenon reflects interactions between cognitive bias, communication processes, epistemic hierarchy and healthcare system factors that influence diagnostic reasoning. Viewed in this manner, medical gaslighting becomes an observable and potentially measurable vulnerability within existing diagnostic safety frameworks, thereby creating opportunities for targeted quality improvement interventions.

### 3.2. Medical Gaslighting as an Epistemic and Diagnostic Failure

**Figure 1:**
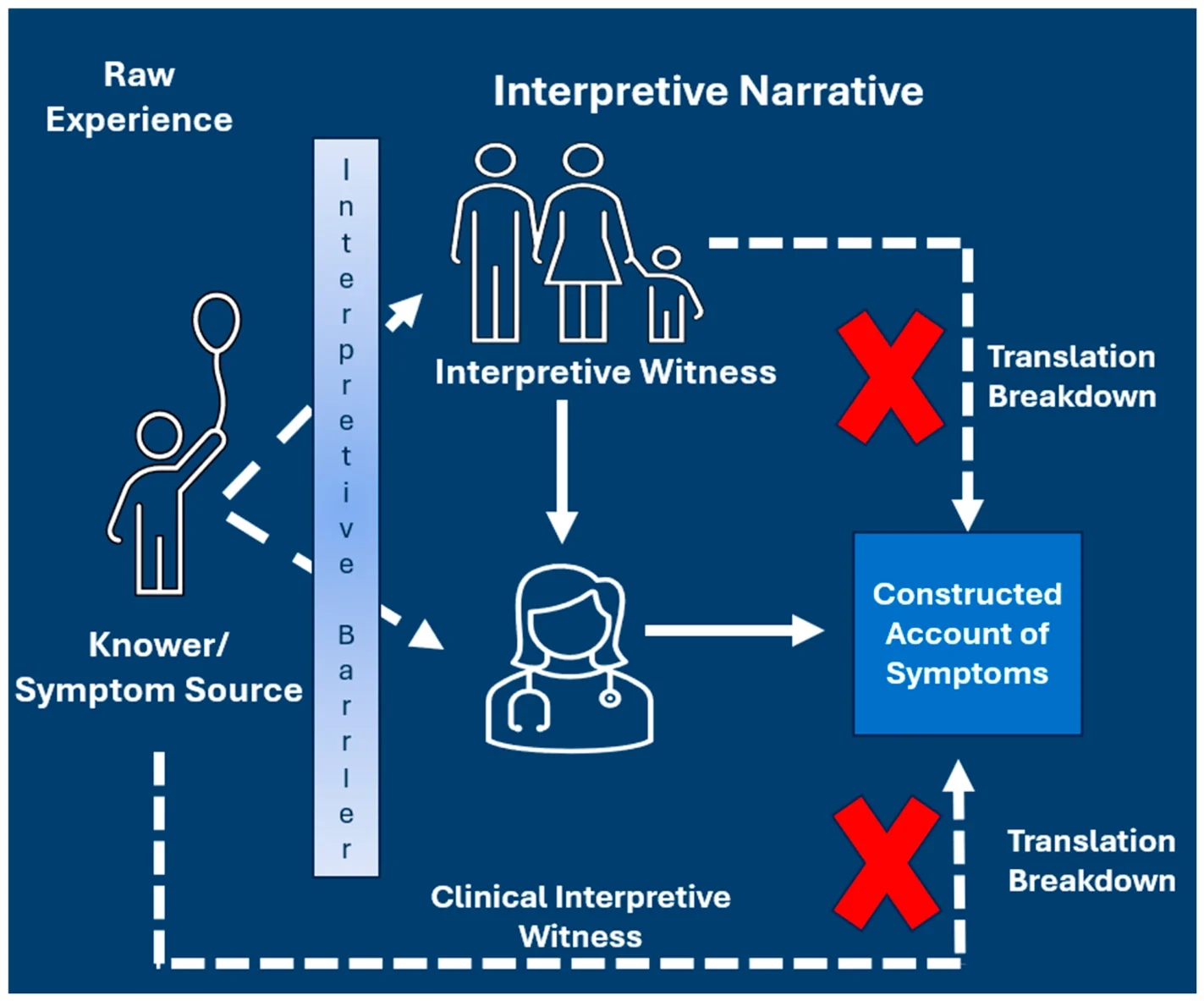
A conceptual mode of communication in pediatric healthcare, illustrating interactions among the child, caregiver, and provider. The child directly experiences symptoms and depending on developmental capacity serves as the primary source of that experience. Caregivers and providers act as interpretive witnesses working together and independently to translate the child’s account into a clinical narrative. This translation produces potential distortion, creating diagnostic risk, and patient safety vulnerabilities when patient and caregiver perspectives are incompletely or inaccurately incorporated into the provider’s assessment.

Medical gaslighting can also be understood through the framework of epistemic injustice, a concept describing harm that occurs when individuals are discredited as reliable knowers of their own experiences [9]. Within healthcare, patients possess unique knowledge regarding subjective symptoms including pain, dizziness, fatigue and functional changes. In pediatric care, however, these experiences are frequently communicated indirectly through caregivers before being interpreted by clinicians (Figure 1).

Each stage within this communication pathway presents opportunities for information loss, reinterpretation or unintentional discounting. As information passes from child to caregiver and subsequently to the clinician, discrepancies may emerge between the patient’s lived experience and the clinical narrative ultimately documented in the medical record. Even when clinicians act in good faith, age-related power differentials, implicit bias, competing diagnostic priorities, and reliance on objective findings may unintentionally diminish the epistemic authority of children and caregivers.

In practice, this may manifest as greater emphasis on laboratory investigations or imaging studies than on persistent patient-reported symptoms. While objective findings remain fundamental to medical diagnosis, disproportionate weighting of measurable data may unintentionally reduce consideration of clinically meaningful subjective information, especially during early or evolving disease.

Epistemic discounting therefore represents more than a communication challenge; it constitutes a potential diagnostic safety vulnerability when it alters the weighting of clinical information used during diagnostic reasoning.

These vulnerabilities can be operationalized as observable process failures including premature diagnostic closure despite persistent symptoms; inadequate documentation of caregiver concerns; failure to reconsider diagnostic hypotheses when clinical trajectories change; absence of explicit contingency planning; and insufficient reassessment following negative initial investigations.

Within the National Academy of Medicine’s diagnostic process framework, these failures correspond to identifiable domains of information gathering, interpretation, communication, and follow-up [5]. Consequently, they provide measurable targets for diagnostic quality improvement rather than remaining solely subjective patient experiences.

### 3.3. Cognitive Bias, Diagnostic Overshadowing, and Premature Closure

Medical gaslighting frequently emerges through the cumulative effects of cognitive bias rather than isolated clinical error. Anchoring bias, framing effects, confirmation bias, and premature closure may all influence diagnostic reasoning, particularly when initial evaluations appear reassuring or when previous diagnostic labels strongly influence subsequent clinical encounters [3,4].

Diagnostic overshadowing represents one of the most important contributors to delayed diagnosis. New or evolving symptoms may be attributed to pre-existing psychiatric illness, neuro-developmental disorders, chronic disease, or medically unexplained symptoms without sufficient re-consideration of alternative diagnoses [10]. Although these cognitive shortcuts may improve efficiency under routine circumstances, they may also increase susceptibility to diagnostic error when evolving clinical information is insufficiently incorporated into ongoing reasoning.

These cognitive mechanisms are well-established contributors to diagnostic error and should therefore be understood as patient safety vulnerabilities rather than isolated communication failures.

Concerns regarding rare conditions such as factitious disorder imposed on another require diagnostic caution. While clinicians must remain vigilant when safeguarding children, premature attribution of deceptive intent may itself function as a powerful anchoring bias that narrows diagnostic reasoning and limits subsequent consideration of alternative explanations. From a systems perspective, unchecked anchoring effects may progressively constrain diagnostic hypothesis generation, reduce openness to new information and delay recognition of evolving disease.

Structured diagnostic reflection, cognitive forcing strategies and deliberate incorporation of patient- and caregiver-reported information represent practical approaches to mitigating these biases and improving diagnostic safety.

### 3.4. Pediatric Vulnerability and Diagnostic Risk

Children are uniquely vulnerable to diagnostic error because accurate diagnosis often depends on interpretation of caregiver-mediated histories, evolving symptom patterns and developmental context rather than direct patient communication alone. Unlike adults, children may have a limited ability to describe the onset, quality, severity or progression of symptoms, requiring clinicians to integrate information from multiple sources while accounting for age-related developmental differences, Consequently diagnostic accuracy depends not only on disease presentation but also on the completeness and interpretation of information exchanged between children, caregivers, and clinicians (Figure 1).

Figure 1 illustrates this multistep communication pathway, and highlights points at which clinically important information may be unintentionally lost, discounted or reinterpreted before being incorporated into diagnostic reasoning.

Because pediatric diagnosis relies heavily on caregiver observations, clinician interpretation of caregiver credibility becomes an important component of diagnostic decision- making. Persistent caregiver advocacy following negative investigations or inconclusive findings may occasionally be interpreted as anxiety, exaggeration, or excessive concern rather than recognition of evolving disease [8]. Although these interpretations are rarely intentional, they may reflect cognitive processes such as premature diagnostic closure, confirmation bias, or diagnostic overshadowing that influence subsequent diagnostic reasoning [2,3,4,10].

These vulnerabilities are particularly important because many pediatric disorders evolve gradually and may initially present with intermittent, nonspecific, or subjective symptoms. Early manifestations of neurological, autoimmune, metabolic, endocrine and inflammatory disorders frequently lack definitive objective findings during initial clinical evaluation. As diseases progress, however, previously isolated symptoms may acquire diagnostic significance that was not apparent at presentation.

This dynamic emphasizes the iterative nature of pediatric diagnosis. Diagnostic reasoning should be viewed as a longitudinal process requiring repeated reassessment as new clinical information becomes available rather than a single decision reached during the initial encounter.

Evidence suggests that evolving or atypical disease presentations contribute substantially to pediatric diagnostic error. Reports indicate that up to one quarter of pediatric intensive care admissions involve children whose conditions evolved in ways that complicated early recognition [2,7,11]. Similar diagnostic challenges have been described in pediatric neurologic disorders, autoimmune diseases, inherited metabolic conditions, endocrine disorders, and inflammatory illnesses where early symptoms are often subtle, fluctuating, or nonspecific [2,7,11].

When clinicians fail to revisit diagnostic hypotheses as symptoms evolve, opportunities for timely diagnosis may be missed. Importantly families may perceive dismissal even when the eventual diagnosis proves benign if communication surrounding diagnostic uncertainty is inadequate. Thus, perceived medical gaslighting may occur independent of diagnostic accuracy and instead reflects failures in communication, validation, and ongoing diagnostic engagement.

Poor communication regarding diagnostic uncertainly has been associated with reduced trust, fragmented care, diminished therapeutic relationships, and increased perceptions of medical error [12]. These downstream effects may contribute to patient safety risks by reducing caregiver willingness to seek reassessment when symptoms worsen or evolve.

### 3.5. Conditions Particularly Vulnerable to Diagnostic Dismissal

Certain pediatric conditions appear especially susceptible to perceived medical gaslighting because they share characteristics that challenge conventional diagnostic reasoning. These disorders, which are meant to be illustrative examples and not an exhaustive list, frequently exhibit fluctuating clinical courses, subjective symptom burdens, limited objective biomarkers, or prolonged diagnostic trajectories, requiring clinicians to rely heavily on patient- and caregiver-reported experiences.

Examples include postural orthostatic tachycardia syndrome (POTS) [13], functional abdominal pain disorders, juvenile fibromyalgia [14], post-acute sequelae of SARS-CoV-2 infection (long COVID) [15], and endometriosis among adolescents [16]. Although these conditions differ substantially in pathophysiology, each presents diagnostic challenges because symptoms may fluctuate over time and objective investigations may initially appear normal or inconclusive.

Children with neurodevelopmental or psychiatric diagnoses may experience additional vulnerability because of diagnostic overshadowing. Existing diagnoses such as autism spectrum disorder, attention deficit hyperactivity disorder (ADHD), anxiety disorders, depression or functional neurologic disorders may inadvertently influence interpretation of new symptoms, increasing the likelihood that emerging physical illness is attributed to pre-existing conditions rather than prompting reconsideration of alternative diagnoses [9,17].

Similarly, rare diseases including mitochondrial disorders, autoimmune diseases, endocrine disorders, genetic syndromes and uncommon inflammatory conditions often fall outside of routine diagnostic frameworks and may therefore experience prolonged diagnostic delays before recognition [18].

Importantly, these examples should not be interpreted as evidence that clinicians intentionally dismiss patients with these conditions. Rather, they illustrate situations in which diagnostic uncertainty, limited objective findings, evolving disease trajectories, and cognitive bias intersect to increase vulnerability to diagnostic process failures.

Across these diverse clinical conditions, a common theme emerges: diagnostic reasoning depends heavily upon effective integration of patient- and caregiver-reported information. When subjective experiences are inadequately incorporated into clinical decision making, families may perceive that their concerns have not been meaningfully considered despite absence of malicious intent.

This perspective reinforces the concept that medical gaslighting represents a system-level diagnostic safety issue rather than solely an interpersonal communication problem.

### 3.6. Consequences for Safety and Quality

The consequences of medical gaslighting extend well beyond dissatisfaction with healthcare encounters. When viewed through the framework of diagnostic safety, failure to adequately recognize, validate or integrate patient- and caregiver-reported information may contribute directly or indirectly to delayed diagnosis, inappropriate diagnostic labeling, fragmented care, increased healthcare utilization and avoidable patient harm [6,7].

Delayed recognition of evolving disease represents one of the most important clinical consequences. Diagnostic reasoning that prematurely converges on an incorrect explanation may reduce subsequent consideration of alternative diagnoses despite persistence or progression of symptoms. This process may lead to repeated healthcare encounters, unnecessary investigations, prolonged diagnostic uncertainty, and delayed initiation of appropriate treatment.

Psychological consequences are also important, particularly during childhood. Repeated experiences of perceived dismissal may undermine children’s confidence in interpreting their own bodily experiences while reducing caregiver trust in healthcare professionals. Over time, these experiences may influence health literacy, future healthcare seeking behavior, treatment adherence, and willingness to report new or evolving symptoms [5,11].

From a diagnostic safety perspective, erosion of caregiver trust represents an important systems-level concern. Families who believe that previous concerns were dismissed may become less likely to seek timely reassessment, less willing to communicate evolving symptoms, or more likely to pursue fragmented care across multiple healthcare systems. Each of these responses may inadvertently reduce the continuity of diagnostic information and increase opportunities for diagnostic error.

Poor communication surrounding diagnostic uncertainty may also compromise the effectiveness of safety-netting strategies. When contingency plans, expected symptom trajectories or explicit return precautions are not clearly communicated, caregivers may be uncertain when worsening symptoms warrant reassessment. Consequently, delayed presentation following clinical deterioration may occur despite appropriate initial management.

These downstream consequences align closely with established mechanisms of downstream error described within the patient safety literature, including failure in information gathering, follow-up, communication, and longitudinal reassessment. Accordingly, the patient experience commonly described as medical gaslighting may be understood not merely as dissatisfaction with clinical interactions, but as a potential manifestation of deficiencies within diagnostic systems themselves.

Recognizing the downstream effects shifts the focus from assigning individual blame toward identifying modifiable diagnostic processes that can be strengthened through quality improvement initiatives, clinician education, communication training, and system redesign.

## 4. Discussion

### 4.1. Clinical Implications for Diagnostic Safety

This narrative review proposes that experiences commonly described as medical gaslighting can be more constructively understood as manifestations of diagnostic safety vulnerabilities rather than solely as interpersonal failures between clinicians and patients. Framing these experiences within established diagnostic safety models shifts the emphasis from assigning blame towards identifying modifiable diagnostic processes that contribute to delayed, missed, or incorrect diagnoses.

The literature reviewed demonstrates that many experiences characterized by patients and caregivers as medical gaslighting correspond to well-recognized contributors to diagnostic error, including cognitive bias, diagnostic overshadowing, premature diagnostic closure, and incomplete integration of patient- and caregiver-reported information. These mechanisms have long been recognized within diagnostic safety research but have rarely been discussed collectively using the language adopted by patients and families.

Pediatric practice provides a particularly important context for this framework because diagnosis depends heavily on caregiver observations, developmental stage, and longitudinal assessment of evolving symptoms. Unlike many adult conditions, pediatric diagnoses frequently develop over many encounters, requiring clinicians to continually reassess previous diagnostic hypotheses as new information emerges. Maintaining diagnostic flexibility while acknowledging uncertainty is therefore essential to reducing preventable diagnostic error.

Importantly, the framework presented here does *not* suggest that clinicians intentionally dismiss patients or caregivers. Rather, it recognizes that diagnostic reasoning occurs within complex clinical environments characterized by time constraints, competing priorities, incomplete information, and unavoidable shortcuts. Understanding these influences provides opportunities for system-level improvement instead of attributing individual blame.

Viewing medical gaslighting as a diagnostic safety vulnerability also creates opportunities for measurement. Process indicators such as documentation of caregiver concerns, reassessment following persistent symptoms, communication of diagnostic uncertainty, and explicit safety-netting instructions may serve as measurable quality indicators that complement traditional measures of diagnostic error.

### 4.2. Practical Interventions for Clinical Practice

Improving diagnostic safety requires interventions that strengthen both clinical reasoning and communication throughout the diagnostic process. Although no single intervention is likely to eliminate diagnostic error, multiple complementary strategies may reduce the likelihood that patient- and caregiver-reported information is inadvertently discounted. Addressing medical gaslighting requires reframing it as a diagnostic safety issue rather than an interpersonal failure. This reframing ensures that interventions target modifiable cognitive and system-level processes within diagnostic care pathways (Table 1).

At the clinician level, deliberate acknowledgement of caregiver concerns, transparent communication regarding diagnostic uncertainty, and structured reassessment of evolving symptoms encourage collaborative diagnostic reasoning while reducing premature closure. Cognitive debiasing strategies including diagnostic reflection, consideration of alternative diagnoses, and deliberate review of discordant clinical findings may further improve diagnostic accuracy, particularly in complex presentations.

Workflow modifications can reinforce these behaviors. Structured documentation of patient and caregiver concerns within the electronic health record, standardized diagnostic ‘pause points’ when symptoms persist despite reassuring investigations, and explicit follow-up plans with pre-defined escalation criteria may improve diagnostic completeness while reducing information loss between encounters [19,20].

Importantly, engaging children and caregivers as active participants in diagnostic reasoning should be viewed not only as an ethical obligation but also as an evidence-informed strategy to improve diagnostic performance under conditions of uncertainty.

### 4.3. Limitations

This review has several limitations. First, it represents a narrative review and conceptual synthesis rather than a systematic review; consequently, literature selection was based on relevance rather than predefined eligibility criteria, and publication bias cannot be excluded. Second, the literature specifically addressing medical gaslighting in pediatric populations is extremely limited. As a result, this review integrates evidence from related disciplines including diagnostic safety, cognitive psychology, epistemic injustice, communication science and patient safety. Third, although the conceptual framework proposed here is grounded in established diagnostic safety theory, empirical studies directly examining medical gaslighting as a measurable diagnostic process remain scarce. The relationship described should therefore be interpreted as hypothesis-generating rather than definitive causal pathways. Finally, perceptions of dismissal are inherently subjective and may occur even when diagnostic management is clinically appropriate. Accordingly, improving communication should complement, rather than replace, rigorous diagnostic evaluation.

### 4.4. Future Research

Several important questions remain unanswered. Development of standardized definitions and validated measures of medical gaslighting would facilitate more rigorous investigation of its prevalence, determinants and relationship with diagnostic outcomes. Future studies should evaluate whether documentation of patient and caregiver concerns, structured communication of diagnostic uncertainty, and implementation of diagnostic pause strategies improve diagnostic accuracy, patient experience, and healthcare utilization.

Prospective studies are also needed to examine how epistemic discounting influences diagnostic reasoning across pediatric subspecialties, emergency medicine, primary care, and inpatient settings. Integration of patient-reported experiences with diagnostic quality metrics may provide new opportunities to identify previously unrecognized vulnerabilities within diagnostic systems.

Finally, implementation science approaches should evaluate the effectiveness of organizational interventions including clinician education, structured cognitive debiasing, enhanced communication training, and electronic health record decision support in reducing diagnostic process failures while maintaining efficient clinical care.

## 5. Conclusions

Medical gaslighting, as conceptualized in this review, is best understood not as an allegation of clinician misconduct but as a potential manifestation of diagnostic safety vulnerabilities arising from the interaction of cognitive bias, communication processes, epistemic hierarchy, and healthcare system factors. In pediatric practice, where symptom reporting is frequently mediated by caregivers and diagnostic uncertainly is common, these vulnerabilities may be amplified, increasing the risk that clinically important patient-reported information is unintentionally discounted during diagnostic reasoning.

Reframing medical gaslighting within established diagnostic safety frameworks translates patient and caregiver experiences into observable diagnostic processes that can be measured, evaluated and improved. This perspective strengthens the connection between patient experience and patient safety by emphasizing information gathering, diagnostic reasoning, communication, follow-up, and longitudinal reassessment of modifiable components of high-quality diagnostic care.

Future work should focus on developing validated measures of communication-related diagnostic failures, evaluating interventions that support structured reassessment and cognitive debiasing, and integrating patient- and caregiver-reported information more effectively into diagnostic decision making. Advancing this work has the potential to improve diagnostic accuracy, strengthen trust between families and clinicians, and reduce preventable harm within pediatric health systems.

## Figures and Tables

**Table 1:** Proposed Diagnostic Safety Interventions to Reduce the Risk of Diagnostic Dismissal.

Diagnostic Safety Domain	Proposed Intervention	Potential Benefit
Information gathering	Structured elicitations and documentation of child and caregiver concerns at every encounter.	Improves completeness and accuracy of diagnostic information.
Clinical reasoning	Diagnostic pause when symptoms persist, evolve, or conflict with the initial diagnosis.	Reduces premature diagnostic closure and anchoring bias.
Communication	Explicit discussion of diagnostic uncertainty and differential diagnosis.	Improves transparency, trust, and shared decision making.
Follow-up	Standardized safety-netting with written return precautions and predefined reassessment triggers.	Facilitates earlier recognition of clinical deterioration.
Electronic health record	Structured recording of caregiver-reported symptoms as discrete clinical data.	Improves longitudinal integration of evolving clinical information.
Cognitive debiasing	Routine use of diagnostic reflection and consideration of alternative diagnoses.	Mitigates cognitive bias and diagnostic overshadowing.
Quality Improvement	Incorporation of patient and caregiver experiences into diagnostic safety review and feedback systems.	Supports organizational learning and continuous improvement.

## Data Availability

No new data were created or analyzed in this study. Data sharing is not applicable to this article.
